# Common connective tissue disorder and anti-cytokine autoantibodies are enriched in idiopathic multicentric castleman disease patients

**DOI:** 10.3389/fimmu.2025.1528465

**Published:** 2025-03-13

**Authors:** Allan Feng, Michael V. Gonzalez, Muge Kalaycioglu, Xihui Yin, Melanie Mumau, Saishravan Shyamsundar, Mateo Sarmiento Bustamante, Sarah E. Chang, Shaurya Dhingra, Tea Dodig-Crnkovic, Jochen M. Schwenk, Tarun Garg, Kazuyuki Yoshizaki, Frits van Rhee, David C. Fajgenbaum, Paul J. Utz

**Affiliations:** ^1^ Department of Medicine, Division of Immunology and Rheumatology, Stanford University School of Medicine, Stanford, CA, United States; ^2^ Institute for Immunity, Transplantation and Infection, Stanford University School of Medicine, Stanford, CA, United States; ^3^ Center for Cytokine Storm Treatment & Laboratory, University of Pennsylvania, Philadelphia, PA, United States; ^4^ Science for Life Laboratory, Department of Protein Science, KTH Royal Institute of Technology, Stockholm, Sweden; ^5^ Myeloma Center, University of Arkansas for Medical Sciences, Little Rock, AR, United States; ^6^ Department of Biomolecular Science and Regulation, The Institute of Scientific and Industrial Research, Osaka University, Osaka, Japan

**Keywords:** iMCD, TAFRO, luminex, protein array, autoantibody, connective tissue disorders, autoimmunity

## Abstract

**Introduction:**

Idiopathic Multicentric Castleman Disease (iMCD) is a polyclonal lymphoproliferative disorder involving cytokine storms that can lead to organ failure and death. The cause of iMCD is unknown, but some clinical evidence suggests an autoimmune etiology. For example, connective tissue disorders (CTDs) and iMCD share many clinical features, and autoantibodies have been anecdotally reported in individual iMCD patients. This study investigates whether common autoantibodies are shared across iMCD patients.

**Methods:**

We assembled custom bead-based protein arrays consisting of 52 autoantigens traditionally associated with CTDs and 38 full-length cytokines and screened serum samples from 101 iMCD patients for IgG autoantibodies. We also screened samples with a 1,103-plex array of recombinant human protein fragments to identify additional autoantibody targets. Finally, we performed receptor blocking assays on select samples with anti-cytokine autoantibodies (ACAs) identified by array.

**Results:**

We found that an increased proportion of iMCD patients (47%) tested positive for at least one CTD-associated autoantibody compared to healthy controls (HC) (17%). Commonly detected CTD-associated autoantibodies were associated with myositis and overlap syndromes as well as systemic lupus erythematosus (SLE) and Sjögren’s Syndrome (SS). ACAs were also detected in a greater proportion of iMCD patients (38%) compared to HC (10%), while the protein fragment array identified a variety of other autoantibody targets. One iMCD sample tested positive for receptor blocking against interferon-ω (IFNω).

**Discussion:**

IgG autoantibodies binding autoantigens associated with common CTDs and cytokines are elevated in iMCD patients compared to HC, suggesting that autoimmunity may be involved in iMCD pathogenesis.

## Introduction

Human herpesvirus-8 (HHV-8)-negative/idiopathic multicentric Castleman disease (iMCD) is a rare, hematologic disorder involving multiple enlarged lymph nodes with characteristic histopathology, cytopenias, and systemic inflammation due to a cytokine storm often including interleukin-6 (IL-6) (1–3). Beyond these unifying features, iMCD is clinically heterogenous and is further categorized into three subtypes: (i) iMCD with thrombocytopenia, anasarca, fever, renal failure/reticulin fibrosis and organomegaly (iMCD-TAFRO), (ii) iMCD with idiopathic plasmacytic lymphadenopathy (iMCD-IPL) involving hypergammaglobulinemia and thrombocytosis, and (iii) iMCD-not otherwise specified (iMCD-NOS) which does not meet the criteria for other subtypes (4). Whereas multicentric Castleman disease (MCD) can be caused by uncontrolled infection with HHV-8 (HHV-8-associated MCD) or monoclonal plasma cells (POEMS-associated MCD), the etiology of all three subtypes of iMCD is unknown.

Several hypotheses for the etiology of iMCD have been proposed including infection, autoimmunity, autoinflammation, and a neoplastic process. Given the heterogeneity of clinical subtypes and variability of response to anti-IL-6 therapy, iMCD pathogenesis may involve multiple mechanisms. Recent studies suggest that an acute infectious etiology is unlikely to be the cause of iMCD and limited genomic studies have failed to identify shared molecular aberrations (5, 6). However, other studies suggest that investigation of autoimmunity may be warranted given anecdotal reports of autoantibodies in iMCD (7–11) and the clinical overlap between iMCD and connective tissue disorders (CTDs) like systemic lupus erythematosus (SLE). In this study, we investigate the role autoimmunity may play in iMCD pathogenesis by quantifying levels of serum immunoglobulin G (IgG) autoantibodies using large scale protein arrays.

## Materials and methods

### Cohort selection and study approval

Samples from iMCD patients were obtained from the University of Pennsylvania (UP, n = 44 samples from 38 patients; six patients with flare/remission samples), University of Arkansas for Medical Sciences (UAMS, n = 51 samples from 45 patients; five patients with flare/remission samples), and Osaka University (OU, n = 26 samples from 18 patients; eight patients with flare/remission samples). Flare and remission designations have been used previously. Disease flare was determined based on clinical features and laboratory test results, including hypoalbuminemia (<3.5 g/dL), elevated CRP (>10 mg/L), anemia (hemoglobin < 13.5 g/dL), renal dysfunction (creatinine > 1.3 mg/dL), constitutional symptoms, and fluid accumulation. Remission was defined as CRP < 10 mg/L, albumin > 3.5 g/dL, hemoglobin > 11.5 g/dL and currently not undergoing hospitalization (12). Hodgkin Lymphoma (HL) samples (n = 20) were obtained from UP. HC (n = 30) were obtained from Stanford Blood Bank and Stanford Hospital, UP, and OU. Five positive control plasma samples from patients with CTDs were purchased from Immunovision, while the positive control sample with anti-IFNγ autoantibodies was collected from a patient with atypical mycobacterial infection (AMI) at Stanford Hospital. Serum samples from all centers were obtained following informed consent and IRB approval (Penn cohort: IRB# 824758, Osaka University: IRB# 2021390, University of Arkansas for Medical Sciences: IRB# 249901).

### Bead-based antigen arrays

Two custom antigen panels were used to generate bead arrays as previously described (13–20). The CTD array consisted of 52 antigens associated with categories of traditional CTDs including Scleroderma, Myositis/Overlap Syndromes, SLE/Sjögren’s Syndrome (SS), gastrointestinal(GI)/Endocrine, DNA-Associated, and Inflammation/Stress. The ACA array included 38 cytokines and cell surface proteins. Briefly, antigens were coupled to color-coded carboxylated magnetic beads (MagPlex, Luminex Corp.). 8 μg of antigen or control antibody was diluted in phosphate-buffered saline (PBS) and transferred to 96-well plates. One bead ID was processed without coupling to any antigen to quantify bead surface binding. Beads were distributed into 96-well plates (Greiner BioOne) and washed in PBS using a 96-well plate washer (Biotek). Each bead was activated by incubating with 0.5 mg 1-ethyl-3(3 dimethylaminopropyl)carbodiimide (Pierce) and 0.5 mg N-hydroxysuccinimide (Pierce) in 100 μl of phosphate buffer for 20 min. After incubation, beads were washed and resuspended in activation buffer (0.05 M 2-N-Morpholino Ethane Sulfonic acid, MES, pH 5.0). Diluted antigens and control antibodies were incubated with beads for 2 hours at room temperature. Beads were washed three times in 100 μl PBS-Tween, re-suspended in 60 μl storage buffer (Blocking reagent for Enzyme-Linked Immunosorbent Assay, ELISA, Roche), and stored at 4°C. Prototype human plasma samples were used for validation of bead arrays. Beads were combined in storage buffer (Blocking reagent for ELISA, Roche) to create each respective array. A comprehensive list of antigens, vendors, and catalogue numbers is in **Supplementary Tables S1, S2**.

To explore a broader set of antigens, a custom human protein fragment array (25-150 amino acids) was constructed in collaboration with the KTH Royal Institute of Technology as previously described (21) and similarly to the full-length CTD and ACA arrays with minor changes. In short, protein epitope signature tag (PrEST) antigens were coupled to carboxylated magnetic beads (MagPlex-C, Luminex Corp.), which were distributed in 96-well plates (Greiner BioOne, Longwood, FL), washed and re-suspended in phosphate buffer (0.1 M NaH2PO4, pH 6.2) using a plate magnet (Dexter) and a plate washer (EL406, Biotek, Winooski, VT). Beads were activated with 0.5 mg 1-ethyl-3(3 dimethylaminopropyl)carbodiimide (Pierce) and 0.5 mg N-hydroxysuccinimide (Pierce) in 100 μl phosphate buffer and incubated for 20 min incubation on a shaker (Grant Bio). After washing antigens were added at a concentration of 40 μg/ml in activation buffer. Activated beads and antigens were incubated together for coupling for 2 hours at room temperature. Conjugated beads were washed in PBS-T and then combined in storage buffer (Blocking reagent for ELISA, Roche).

### Array probing

Serum samples were diluted 1:100 in 0.05% PBS-Tween supplemented with 1% (w/v) bovine serum albumin and transferred into 96-well plates. 5 µl of bead array were distributed into each well of a 384-well plate (Greiner BioOne). 45 µl of 1:100 diluted sera were transferred in duplicate into the 384-well plate containing the bead array. Samples and beads were incubated for 60 min on a shaker at room temperature and then washed with 3 × 60 µl PBS-Tween on a plate washer (EL406, Biotek). 50 µl of 1:1000 diluted R-phycoerythrin (R-PE) conjugated Fc-γ-specific goat anti-human IgG F(ab’)2 fragment (Jackson ImmunoResearch) were added to each well for detection of bound human IgG. After 30 min of incubation with the secondary antibody, the plate was washed with 3 × 60 µl PBS-Tween and re-suspended in 50 µl PBS-Tween. Samples were analyzed using a FlexMap3D™ instrument (Luminex Corp.). Binding events were measured as Median Fluorescence Intensity (MFI).

### Cell-based receptor blocking assays

Serum samples positive by array for anti-IFNα2 and anti-IFNγ were assessed using blocking assays and U937 (ATCC CRL1593) cell lines as previously described (22, 23). Cells (400,000 cells/condition) were incubated with media, 10% HC serum, 10% patient serum, or blocking serum from a prototype patient with atypical mycobacterial infection (AMI) for 15 mins. Cells were then stimulated with 2.5 ng/mL of either IFNα2 (HumanKine, HZ-1066) or IFNγ (HumanKine, HZ-1301). Cells were analyzed on a BD LSR-II flow cytometer, and FACS data were analyzed using FlowJo.

To evaluate the blocking activity of anti–IFNλ2, anti–IFNβ, anti–IFNω, and anti-IL-31 autoantibodies, we used SEAP-reporter HEK293 cell lines stably expressing human receptors specific to these antigens, along with necessary signaling proteins. For TNFα blocking assays, a luciferase-reporter HVEM-293 cell line was employed. We determined the effective concentration (EC75) of the antigens and used this concentration at 10% in a mixture with either 10% healthy control serum or patient serum/plasma, making up a total volume of 50 µL with the cells. Blocking activity was measured by absorbance at 620 nm using a BioTek Synergy HTX Multimode Reader. The results were normalized to the negative controls (cells with only antigen, without any antibody) to demonstrate the antigen stimulation index as an inverse representation of blocking activity. For samples showing blocking activity, additional serum dilutions were prepared to identify the endpoint of the blocking effect.

### Clinical-grade autoantibody assays

Data from clinical grade assays were extracted from medical records and performed by diagnostic laboratories (primarily Quest Diagnostics and Labcorp) as part of clinical evaluation and diagnosis. A list of clinical tests and associated catalogue numbers are available in **Supplementary Table S3**. The clinical autoantibody assays (e.g., Anti-nuclear antibody, ANA titers) evaluated are expressed as a ratio of a serial dilution indicating the amount of a given antibody in a sample.

### High throughput protein quantification

Somalogic’s SOMAscan technology was used to quantify 6,383 proteins in serum from select iMCD patients in the UP cohort (n = 10). Proteins overlapping between the bead-based arrays and SOMAscan were used to investigate quantitative differences between samples with positive and negative autoantibody signals (24).

## Extraction of CHA score as a measure of disease severity

A C-reactive protein (CRP), Hemoglobin (Hgb), and Albumin (Alb) score was calculated for each iMCD patient as a measure of clinical severity and disease activity (CHA score) (25). Peak values were selected for inclusion in the CHA score regardless of lab test timing.

### Statistical analysis

R v4.2.2 and RStudio v2023.06.2 were used to perform analyses (26, 27). MFI values were normalized by subtracting MFI for “bare bead” IDs from MFI values for conjugated bead IDs. Replicate MFI values were averaged.

Autoantibody testing in a clinical setting typically involves setting a standard cutoff for what is considered “positive” for each antigen. In many cases, this is set at a level of at least 3 standard deviations (SD) above the mean for healthy controls who are known to not have this reactivity. We elected to use a much more stringent 5SD above the HC mean cutoff to reduce false positivity rates for individual autoantibodies. Highly-stringent cutoffs also facilitate identification of samples with higher levels of autoantibodies that may be prioritized for mechanistic studies such as the ACA neutralization assays in this study. Thus, samples were considered “positive” for autoantibodies if normalized MFI was greater than 5SD above the average MFI for HC and greater than 3,000 MFI (14). Samples run on the protein fragment array were considered autoantibody-positive if the normalized MFI value was >5SD above the HC mean and greater than 500 MFI. Patients with longitudinal samples were deemed autoantibody-positive if the mean MFI of all timepoints was >5SD above the HC mean and MFI was >3,000.

Statistical differences in MFI were determined using two-sided Wilcoxon rank-sum tests with Bonferroni correction for multiple comparisons. Statistical differences in MFI between paired samples was determined using one-tailed signed rank tests. Fisher’s exact test was used to determine associations between autoantibody positivity and disease status. Spearman’s correlation was used to determine the relationship between CHA score and autoantibody prevalence. A Fisher’s exact test was used to compare overall prevalence of autoantibodies between experimental groups. GraphPad Prism was used for dot and line plots. R package Complexheatmap was used to visualize heatmaps (28).

## Results

### Autoantibodies targeting autoantigens traditionally associated with CTDs are elevated in iMCD

We investigated autoantibody prevalence in six categories of CTD antigens: Scleroderma, Myositis/Overlap Syndromes, SLE/SS, GI/Endocrine, DNA-Associated, and Inflammation/Stress across three cohorts of iMCD patients. There were no sex-based differences (p = 0.38) between cohorts, but there were statistically significant differences between cohorts for ethnicity, age, iMCD subtype, and therapy prior to blood draw (**Table 1**).

**Table 1:** Clinical characteristics of iMCD patients from the University of Pennsylvania (UP), University of Arkansas for Medical Sciences (UAMS), and Osaka University (OU) cohorts.

	UP* (n = 37)	UAMS^ (n = 45)	OU (n = 18)	P-values
**Sex [Percent; (N)]**				0.38
Female	51.3% (19)	40.0% (18)	33.3% (6)	
Male	48.7% (18)	60.0% (27)	66.7% (12)	
**Self-reported ethnicity [Percent; (N)]**				<0.05
White	70.3% (26)	75.6% (34)	0.0% (0)	
Asian Indian	2.7% (1)	2.2% (1)	0.0% (0)	
American Indian or Alaska Native	5.4% (2)	0.0% (0)	0.0% (0)	
Filipino	2.7% (1)	0.0% (0)	0.0% (0)	
Black or African American	2.7% (1)	13.3% (6)	0.0% (0)	
Chinese	5.4% (2)	2.2% (1)	0.0% (0)	
Japanese	0.0% (0)	0.0% (0)	100.0% (18)	
Other	8.1% (3)	6.7% (3)	0.0% (0)	
Refuse to Answer	2.7% (1)	0.0% (0)	0.0% (0)	
**Age at diagnosis [Median (IQR)]**	37.9 (25.6-47.8)	45.7 (35.5-57.3)	56.5 (36.3-63.0)	<0.05
**Subtype [Percent; (N)]**				<0.05
TAFRO	56.8% (21)	28.9% (13)	0.0% (0)	
IPL	10.8% (4)	17.8% (8)	5.6% (1)	
POEMS	2.7% (1)	0.0% (0)	0.0% (0)	
NOS	29.7% (11)	53.3% (24)	94.4% (17)	
**Autoimmune comorbidity [Percent; (N)]**	10.8% (4)	13.3% (6)	NA	1
**Therapy prior to blood draw [Percent; (N)]**				<0.05
Siltuximab	51.2% (22)	27.5% (14)	0.0% (0)	
Tocilizumab	23.3% (10)	17.6% (9)	50.0% (13)	
Rituximab	48.8% (21)	23.5% (12)	0% (0)	
Chemotherapy	27.9% (12)	21.6% (11)	0.0% (0)	
Untreated	14.0% (6)	27.5% (14)	50.0% (13)	
**Flare/Remission [Percent, (N)]**				0.56
Flare	41.9% (18)	37.3% (19)	50.0% (13)	
Remission	58.1% (25)	62.7% (32)	50.0% (13)	

*In the UP cohort, calculations were based on 37 of 38 total subjects as data was unavailable for one subject. ^In the UAMS cohort, age at sample collection was used as age at diagnosis was not available.

**Figure 1:**
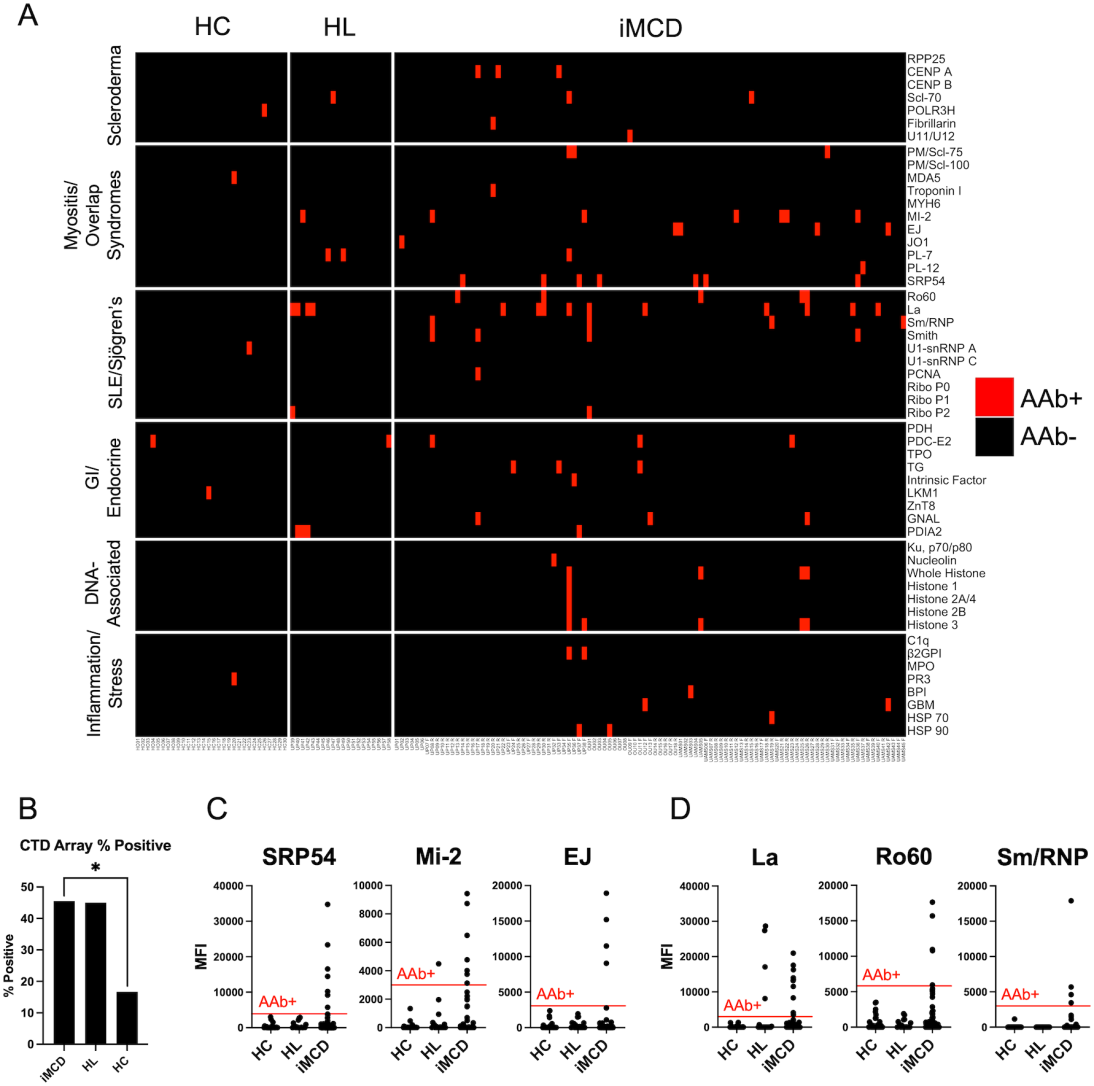
Autoantibodies associated with CTDs are prevalent in iMCD patients. **(A)** Heatmap displaying serum IgG AAbs identified by a 52-plex array of autoantigens associated with traditional CTDs in iMCD patients (n = 101), healthy controls (HC, n = 30) and Hodgkin’s Lymphoma (HL, n = 20) samples. **(B)** Proportion of samples that were positive for at least one autoantibody target in the CTD array (* = *P* < 0.05). A Fisher’s exact test was used to determine significance between groups. **(C)** Dot plots comparing MFI values for three of the most targeted autoantigens associated with myositis and overlap syndromes in iMCD patients. **(D)** Dot plots displaying three of the most commonly targeted autoantigens associated with SLE and SS in iMCD patients.

Overall, significantly more iMCD patients were positive for autoantibodies targeting at least one autoantigen on the CTD array (n = 47 of 101, 47%) than HCs (n = 5 of 30, 17%; *P* = 0.003; **Figures 1A, B**, **Supplementary Figures S1, S2A**). The most frequently detected CTD-associated autoantibodies in iMCD patients were associated with Myositis/Overlap Syndromes (n = 22, 22%; *P* = 0.026) and SLE/SS (n = 18, 18%; P = 0.073), although they did not reach statistical significance compared to HCs after Bonferroni correction. The most common Myositis/Overlap Syndrome autoantibodies in iMCD patients were anti-signal recognition particle 54 (SRP54; n = 7, 7%), anti-Mi-2 (n = 6, 6%), and anti-EJ (n = 4, 4%) (**Figure 1C**). Most SLE/SS-associated autoantibodies detected in iMCD patients targeted La (n = 10, 10%), Ro60 (n = 5, 5%), and Sm/RNP (n = 4, 4%) (w 1D). None of these autoantibodies were detected in HCs. Autoantibodies binding DNA-associated proteins, such as histones and nucleolin, were also found in six (6%) iMCD patients but not in HCs (**Figure 1A**, fifth horizontal panel). When comparing iMCD and HL, which has been previously found to have increased autoantibodies compared to HCs and solid malignancies (33), there were similar proportions of CTD-associated autoantibody positive patients (**Figure 1B**). However, several autoantibodies found in high frequencies in iMCD samples (e.g., anti-SRP54, anti-EJ, anti-Ro60, anti-Sm/RNP) were not detected in any HL samples (**Figures 1C, D**). We also screened five plasma samples from patients with CTDs using the CTD array with more focused antigen content and detected specific autoantibodies associated with each patient’s CTD (**Supplementary Figure S3A**). Taken together, a variety of autoantibodies are more commonly identified in iMCD than HC, and most of them are associated with SLE, SS, myositis, and overlap syndromes.

### Clinical-grade autoantibody lab tests correlate with the research-grade CTD array

34 samples from 29 iMCD patients from the UP cohort were also screened with a clinical-grade ANA (Anti-nuclear antibody) test or other clinical-grade autoantibody screened for by the CTD array (Ro, La, Sm, and RNP) as part of routine clinical care. Results from clinical-grade assays largely correlated with those from the CTD array (**Supplementary Table S4**). **Supplementary Table S4** shows direct comparisons between clinical autoantibody tests and array based results. Concordance was observed in 15/16 patients (94%) for anti-SSA, 12/14 patients (86%) for anti-SSB, and 9/9 patients (100%) for anti-Smith and anti-RNP assays.

### Prevalence of clinical autoantibodies in iMCD

To further interrogate autoantibody burden in iMCD, we queried the ACCELERATE registry (29) to identify 89 iMCD patients who had clinical autoantibodies measured. Thirty-five patients (38%) had a positive ANA result (**Table 2**), and ANA titers, when available, were generally mild-to-moderately elevated across iMCD. No significant differences were observed between clinical subtypes (iMCD-TAFRO, iMCD-NOS, iMCD-IPL, **Supplementary Figure S4**). Among other autoantibodies, for which there were >30 patients assessed, a notable proportion were positive (e.g., anti-SSA, 22%; anti-SSB, 15%; anti-dsDNA, 6%; anti-Sm, 6%; Direct Coombs test, 44%, **Table 2**). A similar prevalence of these autoantibodies was found among the UP cohort of patients who were tested for autoantibodies by clinical-grade assays and research-grade CTD and ACA arrays (**Table 2**).

**Table 2:** Autoantibody clinical assay results in ACCELERATE iMCD patient cohort.

Clinical Assay	Num Pos - Overall	Num Neg - Overall	% Pos -Overall	Num Pos -UP Cohort	Num Neg - UP Cohort	% Pos– UP Cohort	% Consistent between Array and Clinical
**ANA (Qualitative)**	35	54	38	9	13	41	–
**Anti-SSA**	9	32	22	3	10	23	94 (15/16)
**Anti-SSB**	6	34	15	2	10	17	86 (12/14)
**Anti-dsDNA**	3	48	6	1	12	8	–
**Anti-RNP**	1	37	3	0	8	0	100 (9/9)
**Anti-SM**	2	34	6	0	8	0	100 (9/9)
**Direct Coombs (poly)**	14	18	44	2	7	22	–

### Prevalence of autoantibodies targeting secreted and cell-surface proteins in iMCD

Next, we measured ACAs in iMCD patients using a 38-plex array. We first investigated anti-IL-6 antibodies given that a large portion of iMCD patients with known anti-IL-6 therapy status (n = 76) were on anti-IL-6 therapy with siltuximab or had received siltuximab within a year of serum collection (n = 30, 39%). As expected, a high proportion of iMCD patients on siltuximab (n = 27, 90%) tested positive for anti-IL-6 by the ACA array. Anti-IL-6 was then removed from further analyses.

**Figure 2:**
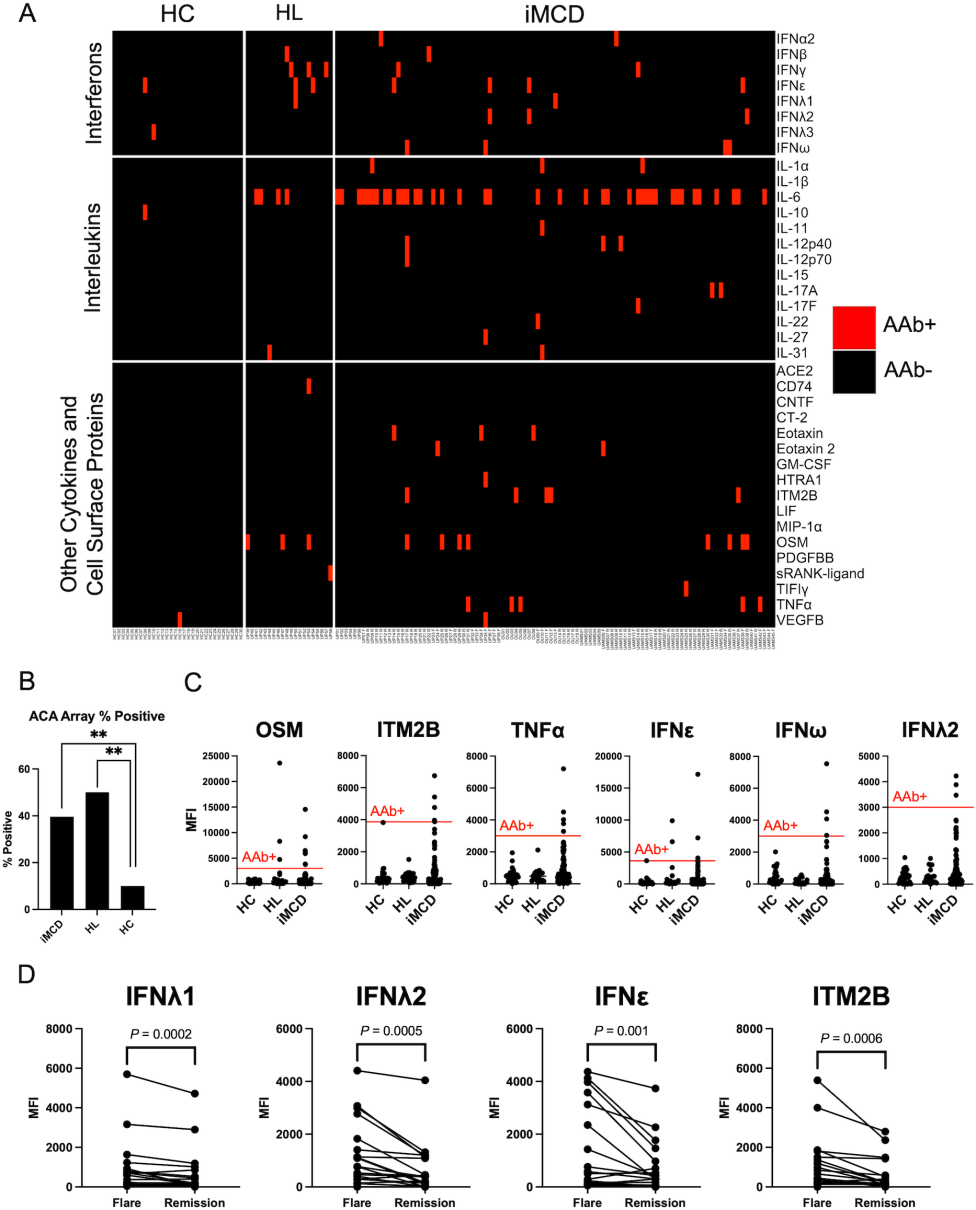
IgG ACAs are common in iMCD patients. **(A)** Heatmap displaying serum IgG ACAs identified by a 38-plex array of secreted and cell-surface proteins in iMCD patients (n = 101), healthy controls (HC, n = 30) and Hodgkin’s Lymphoma (HL, n = 20) samples. **(B)** Proportion of samples that were positive for at least one ACA. iMCD and Lymphoma cohorts were significantly enriched for ACAs (** = *P* < 0.01) compared to HC. A Fisher’s exact test was used to determine significance between groups. **(C)** Dotplots comparing MFI values for six cytokines and cell surface proteins that were most commonly targeted by ACA in iMCD patients. **(D)** Longitudinal analysis comparing flare and remission disease state MFI values. Only comparisons that surpassed a Bonferroni-corrected *P*-value cutoff after Wilcoxon signed rank tests are shown.

Overall, ACAs were detected in significantly more iMCD patients (n = 38, 38%) than HCs (n = 3, 10%; *P* = 0.004) (**Figures 2A, B**, **Supplementary Figures S2B, S5**). The most common ACAs identified in iMCD bound oncostatin M (OSM) (n = 8, 8%), tumor necrosis factor (TNFα; n = 5, 5%), integral membrane protein 2B (ITM2B; n = 5, 5%), IFNϵ (n = 4, 4%), IFNω (n = 4, 4%), and IFNλ2 (n = 3, 3%; **Figure 2C**). There was no significant difference in the proportions of ACA positive iMCD and HL patients with 10 HL patients (50%) positive for at least one ACA (**Figure 2B**). However, certain ACAs such as anti-ITM2B, anti-TNFα, and anti-IFNλ2 were commonly detected in iMCD patients but in no HL or HC samples (**Figure 2C**). We also screened a positive control sample from a patient with atypical mycobacterial infection (AMI) with known anti-IFNγ autoantibodies, which tested positive for anti-IFNγ by the ACA array as expected (**Supplementary Figure S3B**).

We next investigated whether autoantibody levels were temporally associated with disease flare vs. remission states. Longitudinal flare and remission samples were available for 19 iMCD patients (n = 6, UP cohort; n = 5, UAMS cohort; n = 8, OU cohort). No differences were observed in the CTD array, but several ACAs targeting cytokines including IFNλ1 (*P* = 0.0002), IFNλ2 (*P* = 0.0005), IFNϵ (*P* = 0.001), and ITM2B (*P* = 0.0006) were significantly elevated in flare samples compared to matched remission samples (**Figure 2D**).

As an orthogonal approach to identify antibodies to secreted proteins, we utilized a 1,103-plex array of recombinant human protein fragments representing 556 secreted proteins. Sufficient bead pools allowed for analyses of the two largest US cohorts, UP (n = 38 patients) and UAMS (n = 45 patients), and HCs. Autoantibodies recognizing 36 protein fragments in the array were identified in at least one patient from both cohorts (**Supplementary Figures S6A, B**). The most common autoantibodies detected in iMCD samples but in no HCs included anti-JAM3 (7%), anti-LRG1 (7%), anti-HTRA1 (7%), and anti-AGGF1 (7%) (**Supplementary Figures S6C, D**). Overall, the protein fragment array identified additional candidate antigens, but there was little overlap with the ACA array built on full-length proteins.

### Receptor blocking activity in iMCD sera

Next, we performed receptor blocking activity assays to determine whether the ACAs detected in this study are receptor blocking. Upon stimulation with IFNα2 and IFNy, cells treated with patient or control serum had similar levels of pSTAT1 activation, suggesting that anti-IFNα2 and anti-IFNy present in patient sera from two patients in this study are non-blocking (**Supplementary Figure S7A**). A similar approach involved culturing reporter cell-lines with antigen-serum complexes for 18 hours for TNFα and 24 hours for the other cytokines to assess the blocking activity of antibodies. ACA+ iMCD sera were found to be non-blocking for IFNλ2, IFNβ, IL-31, and TNFα (**Supplementary Figure S7B**). However, one sample exhibited robust blocking activity against IFNω, which continued to display blocking activity with serial dilutions until five-fold dilution (**Supplementary Figure S7C**).

### Autoantibody levels do not correlate with clinical severity or serum protein levels

To determine if detected autoantibodies were correlated with iMCD subtypes, we re-analyzed the autoantibody results after separating patients into iMCD-TAFRO, iMCD-NOS, and iMCD-IPL. Each subtype had significantly higher proportions iMCD patients with CTD-associated autoantibodies and ACAs compared to HC, except for ACAs in iMCD-TAFRO (**Supplementary Tables S5A, B**). To examine whether these autoantibodies correlated with clinical severity, we measured CHA scores in the UP cohort where sufficient data existed (25) and found no correlation between CHA score and number of autoantibody positive iMCD patients (**Supplementary Figures S8A, B**). We also evaluated whether ACA+ patients had increased serum levels of target cytokines. We quantified serum levels of IFNϵ, and Eotaxin-2 from 10 patients and found no trend between presence or lack of ACA and serum cytokine levels (**Supplementary Figure S9**).

## Discussion

A fundamental question in CD research is whether iMCD is an autoinflammatory disease, autoimmune disease, neoplastic disease, infectious disease, or some combination. Our aim was to determine whether autoimmune mechanisms may be involved in iMCD pathogenesis. Autoantibodies commonly found in certain CTDs were identified in a serum-based cohort of iMCD patients. Nearly half (47%) of iMCD patients tested positive for at least one CTD-associated autoantibody compared to only 17% of HCs. Most autoantibodies targeted proteins that complex with RNA and DNA and are linked to SLE and myositis. These nucleic acid-associated proteins contained on the CTD array include spliceosomal RNPs (e.g., Sm/RNP), Ro and La, nucleosomal proteins (e.g., Mi-2), aminoacyl-tRNA synthetases (e.g., EJ and Jo1), and SRP54. Interestingly, many of these autoantibodies were recently detected in patients with acute severe COVID-19 (14), and in pre-pandemic samples from patients with infections due to other pathogens in intensive care units (22). In iMCD patients, the increased prevalence of CTD-associated autoantibodies suggests that autoimmunity may partially explain a range of immune-mediated signs, symptoms, and clinical laboratory test abnormalities observed in iMCD that are also associated with autoimmune disorders such as SLE and myositis. Although our arrays did not contain platelet surface proteins or red blood cell (RBC) antigens, a recent article suggests immune-mediated destruction of platelets and RBCs occurs in some iMCD patients (30).

We also identified several ACAs in iMCD patients. ACAs have been increasingly recognized to be able to modulate autoimmune disease course. Neutralizing ACA against Type I IFNs such as IFNα, IFNβ, and IFNω are found in various autoimmune diseases such as SLE, SS, myasthenia gravis, autoimmune polyglandular syndrome type 1 (APS-1), and immunodysregulation polyendocrinopathy enteropathy X-linked syndrome (IPEX) (31). Interestingly, some ACAs do not neutralize but instead stabilize their cytokine targets as is the case with anti-IL-6 in APS-1 (32) and anti-IL-8 in ARDS (33). The ACAs we evaluated were found to be mostly non-receptor blocking, except for one patient with strong blocking activity against IFNω. The presence of anti-OSM in multiple iMCD samples is noteworthy, as OSM is an inflammatory cytokine from the IL-6 cytokine family. OSM is produced by activated T-cells, neutrophils, macrophages, and dendritic cells (34–37), and plays important roles in hematopoiesis, inflammation, fibrosis, mesenchymal stem cell differentiation, and cancer (38–42). TNF and IL-1β are other pro-inflammatory cytokines elevated in some iMCD patients (43, 44) and both cytokines stimulate IL-6 production (45, 46). Interestingly, we detected anti-TNFα in a high proportion of sera from iMCD patients (6%) but none of the samples displayed TNFα blocking activity.

Levels of several ACAs were elevated in flare versus remission states. We hypothesized that autoantibodies correlated with iMCD disease activity would be expected to be higher during disease flare and lower during remission. In total, we identified 4 ACAs, including anti-IFNλ (1 and 2), anti-IFNϵ, and anti-ITM2B, that were significantly upregulated during disease flare. Type-III interferons are critical for immune defense and promote inflammation in certain contexts. IFNλ is increased in several autoimmune rheumatic diseases, including SLE, and correlates with disease activity (47). Although Type-I IFN signatures have been identified in iMCD patients (12) and are hypothesized to work through a JAK dependent mechanism, Type-III IFNs have not been explored. Importantly, current biologic therapies targeting Type-I IFNs or their receptors do not block effects of IFNλ. Notably, TNF inhibition has been recently proposed as a therapy for iMCD (48).

In addition to helping with understanding of iMCD pathogenesis, this study may shed light on diagnosis. Currently, the diagnostic criteria for iMCD requires exclusion of known infectious, malignant, and autoimmune disorders that clinically mimic iMCD. These include autoimmune/autoinflammatory disorders such as SLE, rheumatoid arthritis (RA), systemic juvenile idiopathic arthritis (sJIA), and autoimmune lymphoproliferative syndrome (ALPS) (1). Importantly, all patients included in this study did not fulfill criteria for any of these CTDs. While we did not detect any autoantibody that was elevated across all iMCD patients to suggest it may be a diagnostic biomarker, future studies may identify autoantibodies that contribute to diagnosis. Moreover, increased levels of autoantibodies identified in this study suggest that autoimmune-mediated mechanisms may play a pathogenic role in a subset of iMCD patients. If autoimmune disease mechanisms drive disease, then treatments targeting CD19-positive cells such as bispecific antibodies and chimeric antigen receptor therapies should be considered.

Our study has several limitations. First, while autoantibodies suggest autoimmune involvement, the presence of autoantibodies does not directly implicate autoimmunity as a pathological mechanism. For example, Hodgkin’s disease and post-infectious etiologies can present with elevated autoantibodies. Autoantibody production could also be a response to hypercytokinemia during disease flares. Second, sample sizes were limited in both cases and healthy controls, especially in the longitudinal samples. Third, we chose one primary method for quantifying autoantibodies among several options. We chose to employ focused, bead-based arrays that would be more clinically valuable as a large majority of the autoantibodies we screened could be assessed by clinical lab assays, making results more rapidly translatable.

In summary, this study confirms the presence of autoantibodies in iMCD, which has been anecdotally reported in case studies (7–11). In fact, findings revealed autoantibodies targeting SLE and myositis-specific autoantigens and cytokines in iMCD. We also demonstrate that levels of specific ACA are increased in flare compared to remission. More research is needed to understand the role of these autoantibodies in iMCD pathogenesis. Future studies require expanded longitudinal sampling to ascertain changes in autoantibody levels between disease states. Further mechanistic work is also needed to understand potential functions of autoantibodies in iMCD.

## Data Availability

Deidentified array data are publicly available on the Gene Expression Omnibus (GEO) database with accession numbers GSE290521, GSE290527, GSE290532, GSE290549, and GSE290594. Code used for data analysis and figure generation may requested from the corresponding authors.
