# Supplementary material for: Common connective tissue disorder and anti-cytokine autoantibodies are enriched in idiopathic multicentric castleman disease patients
Source: Front Immunol. 2025 Mar 13;16:1528465. doi: 10.3389/fimmu.2025.1528465 (PMC11966032; doi:10.3389/fimmu.2025.1528465)
Supplement: Supplementary file 1 [file DataSheet1.docx]

**Supplementary Figure S1: Heatmap of normalized MFI data for the CTD array corresponding to Figure 1**.

**A**

**B**

**Supplementary Figure S2: Clustered heatmaps of (A) CTD and (B) ACA arrays.** Log2FC values of difference from average MFI from healthy control samples. Hierarchical clustering within sub-cohort and autoantibody category was performed to identify tren**Supplementary Figure S3: Autoantibody screens of samples from patients with autoimmune diseases. (A)** Positive control plasma samples from patients with autoimmune diseases (n = 5) and healthy control (HC) samples (n = 20) were screened with a version of the CTD array with more focused autoantigen content. **(B)** A positive control serum sample from a patient with atypical mycobacterial infection (AMI) due to confirmed anti-IFNγ autoantibodies and HC samples (n = 20) were screened with a version of the ACA array with more focused autoantigen content.

**
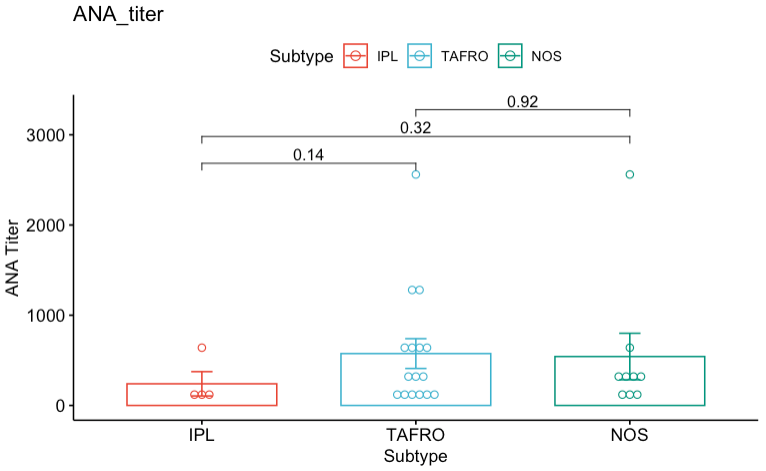
Supplementary Figure S4: Antinuclear Antibody (CTD) titer values in iMCD Clinical Subtypes.** CTD titer values were compared among differing iMCD clinical subtypes. All patients were diagnosed iMCD and subtyped based on previously published criteria. A student’s t-test was used to compare between groups. ANA titer values were compared among differing iMCD clinical subtypes (idiopathic plasmacytic lymphadenopathy, IPL, thrombocytopenia, anasarca, fever, reticulin fibrosis, and organomegaly, TAFRO, and not otherwise specified, NOS. All patients were diagnosed with iMCD and clinically subtyped based on previously published criteria. Antinuclear antibody titers are expressed as a ratio which indicates the concentration of ANA antibodies in a sample. To evaluate numerically, the denominator of each dilution ratio was used (e.g., a ratio of 1:320 would be reflected as 320). A student’s t-test was used to compare between groups. Values above brackets indicate p-values from the indicated comparison.

**Supplementary Figure S5: Heatmap of normalized MFI data for the ACA array corresponding to Figure 2**.


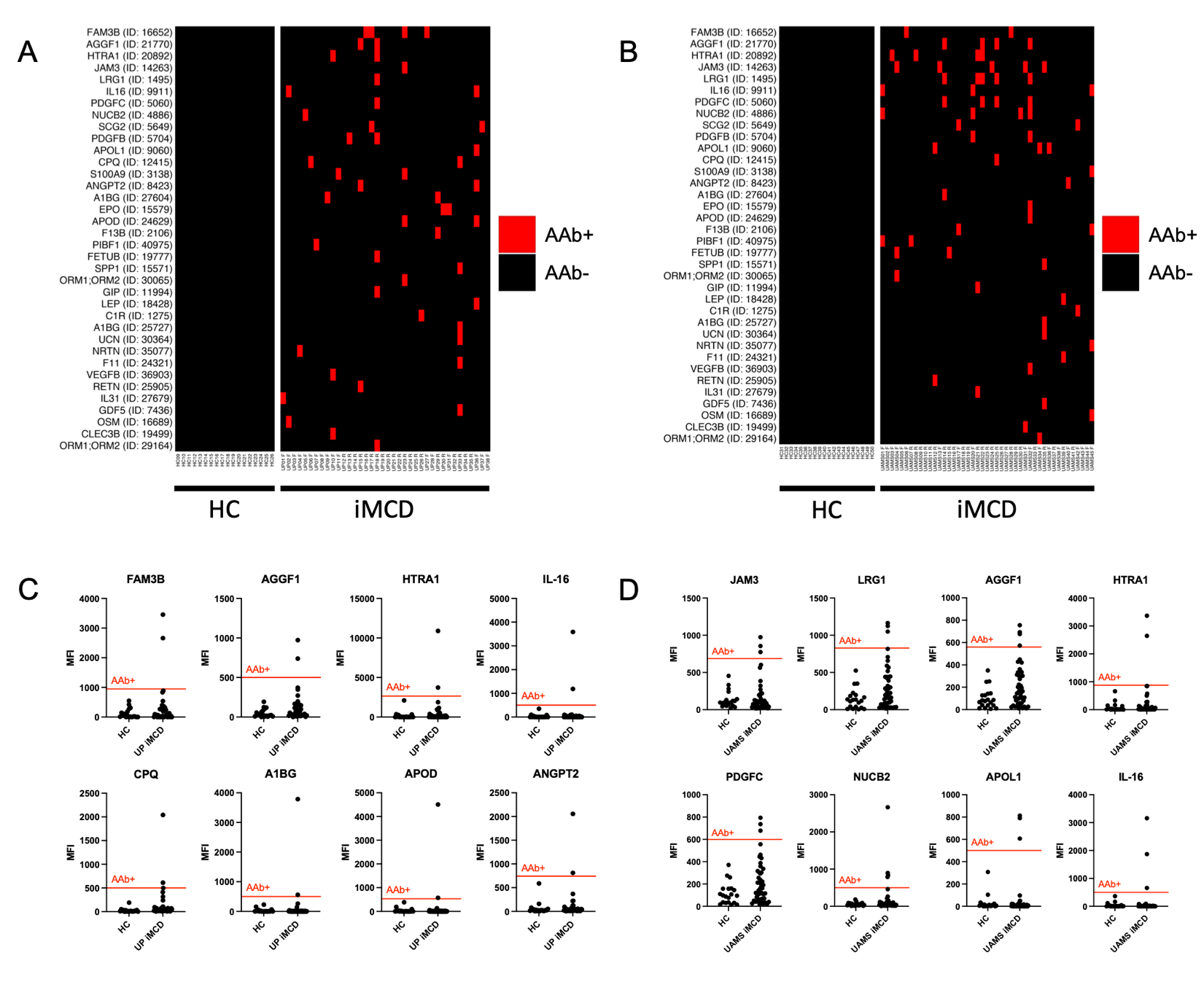


**Supplementary Figure S6.** **Autoantibodies in iMCD recognize fragments of secreted proteins**. **(A)** Heatmap displaying the most commonly targeted PrESTs in the UP cohort. The 1103-plex array of recombinant human Protein Epitope Signature Tags (PrESTs), which are protein fragments of 50 to 150 amino acids, represented 556 secreted proteins selected from the “human secretome”. Serum samples from the iMCD subjects in the UP cohort (n = 38) and HC (n = 18) were screened. Red indicates an autoantibody (AAb) positive hit for a specific antigen and sample. The cutoff for AAb positivity was an MFI value > 5 SD above the average MFI for HC and an MFI value > 500 units. **(B)** Eight representative dot plots displaying MFI values for commonly targeted PrEST antigens in the UP cohort with an autoantibody positivity (AAb+) cutoff denoted by a red line for each antigen. **(C)** Heatmap displaying the most commonly targeted PrESTs in the UAMS cohort. Serum samples from the iMCD subjects in the UAMS cohort (n = 45) and HC (n = 20) were screened. Red indicates an autoantibody (AAb) positive hit for a specific antigen and sample, using the same criteria as previously described **(D)** Eight representative dot plots displaying MFI values for commonly targeted PrEST antigens in the UAMS cohort with an autoantibody positivity (AAb+) cutoff denoted by a red line for each antigen.

**
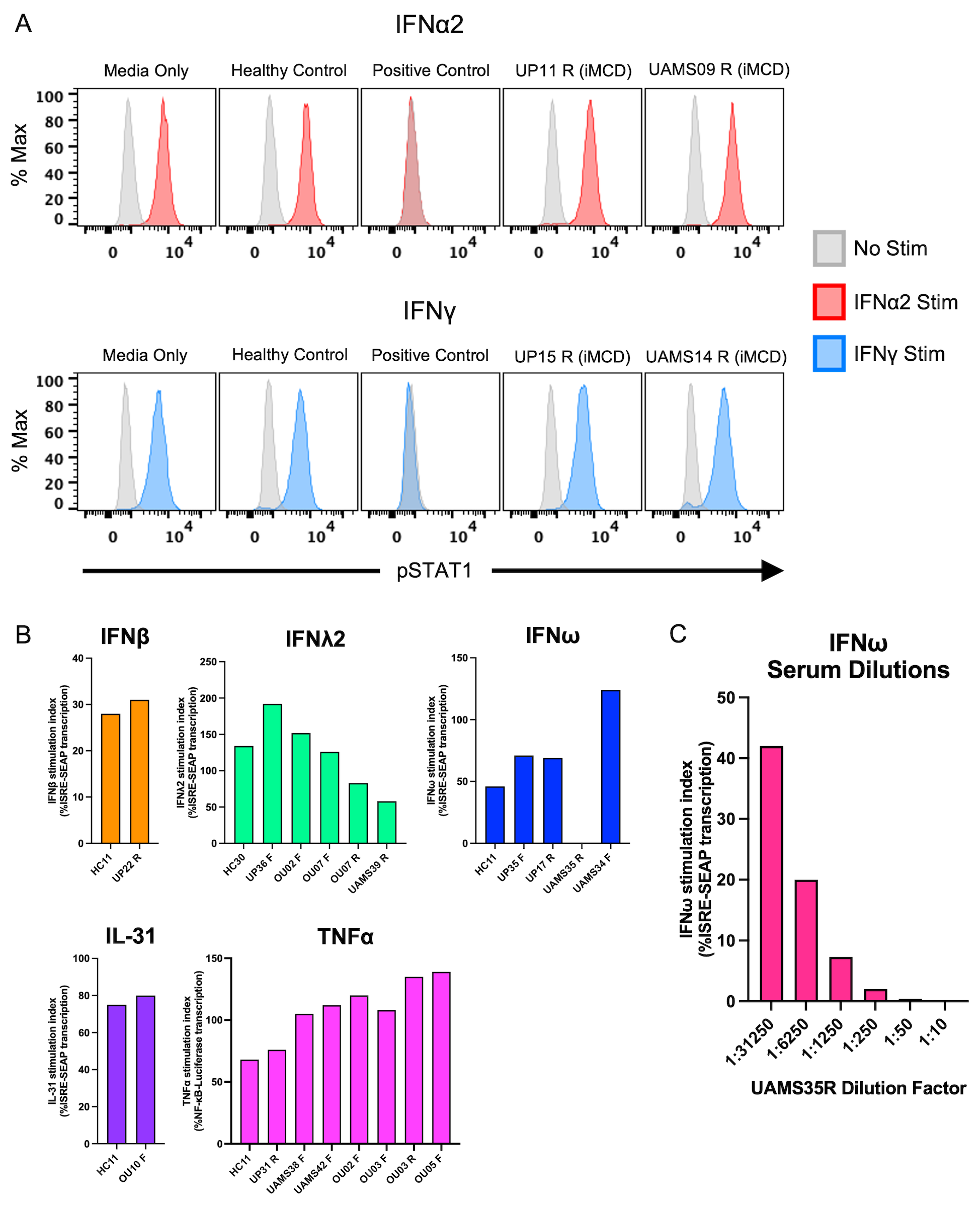
**

**Supplementary Figure S7**: **Receptor blocking activity of iMCD patient sera**. **(A)** FACS plots of cytokine signaling assays for IFNα2 and IFNγ. U937 cells were treated with media only, healthy control (HC) serum, positive control (PC) serum, or iMCD serum that was positive for anti-IFNα2 or anti-IFNγ autoantibodies by array. **(B)** SEAP-based blocking assays for IFNλ2, IFNβ, IFNω, and IL-31 and luciferase-based blocking assay for TNFα. OD620 values were normalized to the negative controls (cells with only antigen and without any antibody). **(C)** SEAP-based blocking using serial dilutions of the IFNω blocking iMCD sample to identify endpoint of the blocking effect.

**A B**

**
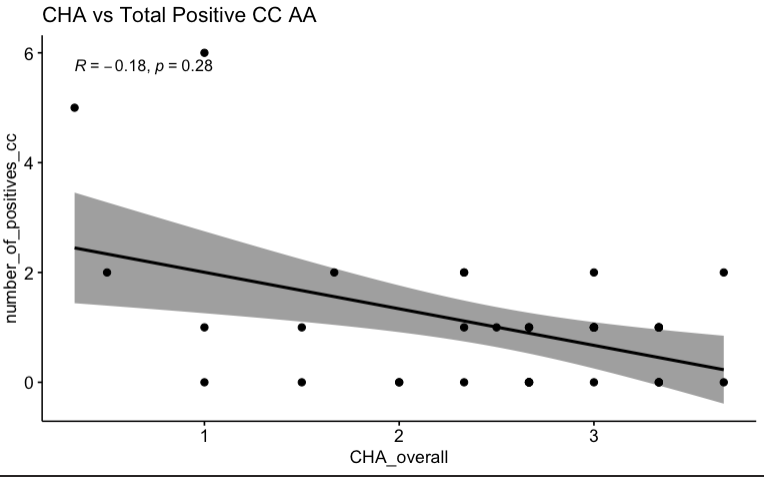

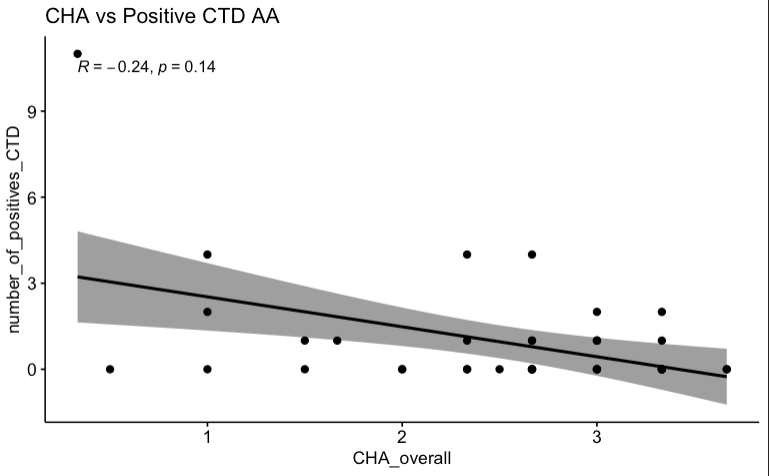
 Supplementary Figure S8: Clinical Severity is not associated with number of positive autoantibodies in the CTD (A) or ACA (B) arrays.** Number of positive autoantibodies on either the CTD or ACA arrays were quantified per patient in the Penn cohort. A CHA score (an index score measuring the severity of disease activity in iMCD) was derived from lab values from each patient. A Pearson’s correlation was performed between both variables.

**
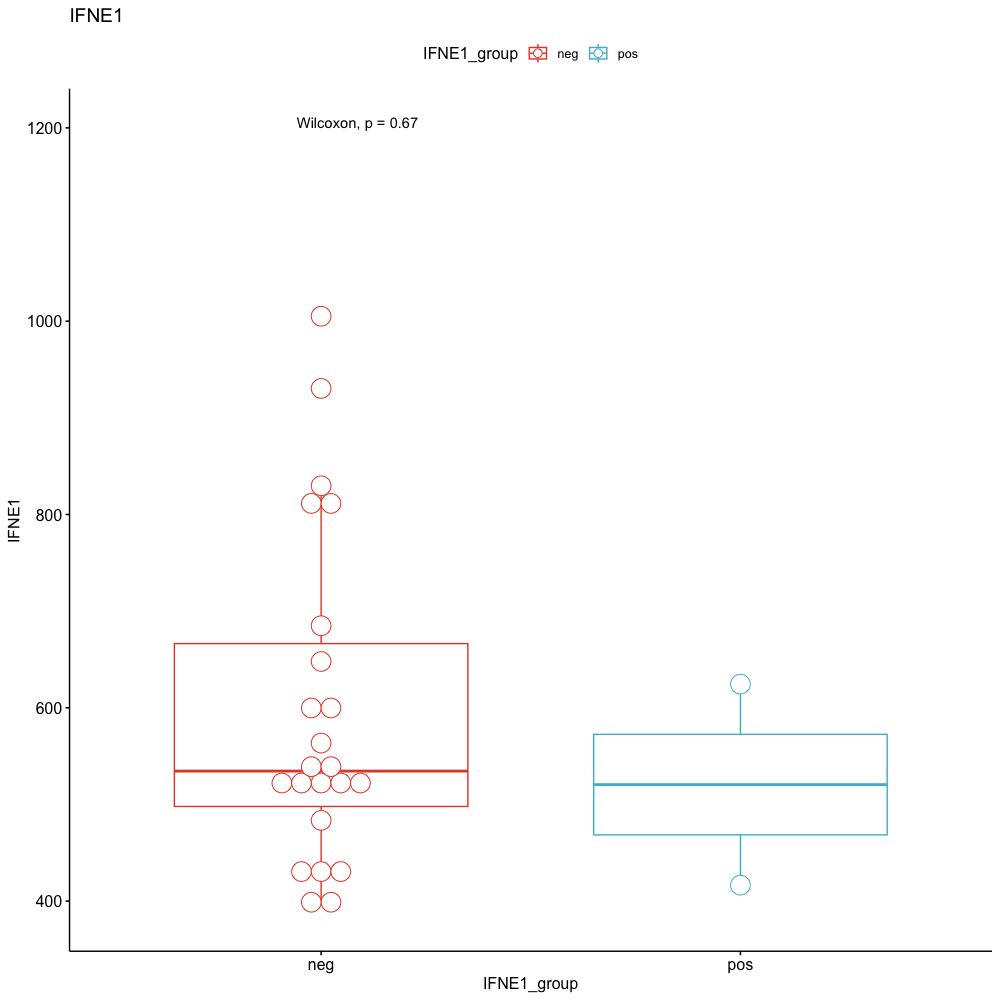
** **
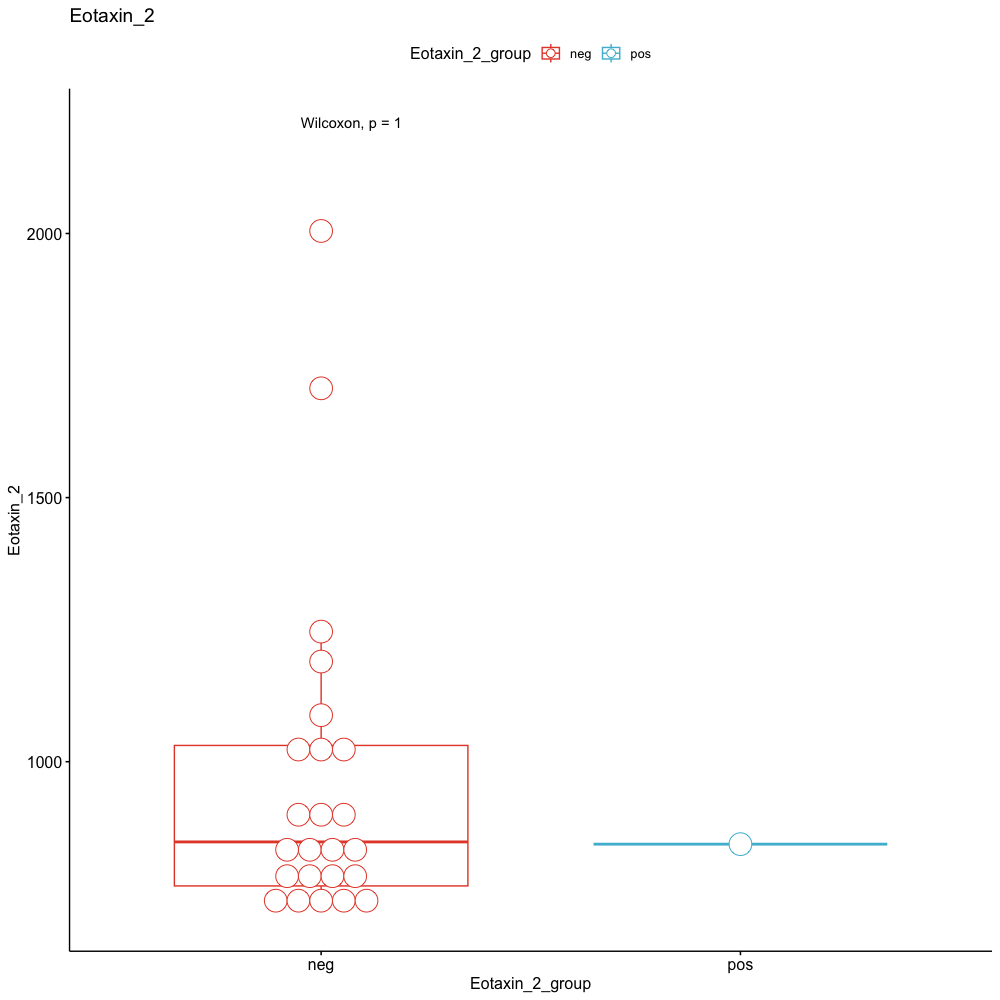
**

**Supplementary Figure S9: Protein analyte quantifications do not show higher levels in subjects with a positive autoantibody signal (mean + 5SD of healthy controls).** The Somalogic Somascan assay was used to quantify protein analytes from serum samples in 10 iMCD patients from the Penn cohort. Relative Fluorescent Untis (RFU) of each protein analyte is shown. A Wilcoxon rank sum test was used to determine significant differences between samples with a ‘positive’ autoantibody and ‘negative’ autoantibody on the CTD or ACA arrays. There was no relationship between positive autoantibody from either array and protein quantification using the Somascan technology.

**Supplementary Table S1. Connective Tissue Disease (CTD) Array content.**

| **Bead ID** | **Antigen** | **Vendor** | **Catalog #** |
| --- | --- | --- | --- |
| 1 | Bare Bead |  |  |
| 2 | Human IgG from serum | Sigma | I4506 |
| 3 | Anti-Human IgG Fc fragment Specific | Jackson | 109-005-008 |
| 4 | Anti-Human IgG (H+L) | Jackson | 109-005-003 |
| 5 | Anti-Human IgG F(ab') fragment specific | Jackson | 109-005-006 |
| 6 | β2GPI | Diarect | A14901 |
| 7 | MPO | Diarect | A18501 |
| 8 | La | Diarect | A12801 |
| 10 | PR3 | Diarect | A18601 |
| 11 | Histone H1 | Immunovision | HIS-1001 |
| 12 | Histone H2A/H4 | Immunovision | HIS-1002 |
| 13 | Histone H2B | Immunovision | HIS-1003 |
| 14 | CENP B | Diarect | A12501 |
| 15 | Histone H3 | Immunovision | HIS-1004 |
| 16 | Whole Histone | Immunovision | HIS-1000 |
| 17 | GBM | Diarect | A16801 |
| 18 | C1q | Biodesign | A90150H |
| 19 | BPI | Arotec | ATB01-02 |
| 24 | Fibrillarin | Prospec | ENZ-566 |
| 31 | U11/U12 | Origene | TP303746 |
| 38 | CENP A | Diarect | A16901 |
| 39 | EJ | Diarect | A11101 |
| 40 | HSP 70 | Stressgen | NSP-555 |
| 41 | HSP 90 | Stressgen | SPP-770 |
| 42 | Intrinsic Factor | Diarect | A16701 |
| 43 | JO1 | Diarect | A12901 |
| 44 | Ku, p70/p80 | Diarect | A17301 |
| 45 | LKM1 | Diarect | A13501 |
| 46 | MDA5 | Diarect | A30001 |
| 47 | MI-2 | Diarect | A18101 |
| 48 | PCNA | Diarect | A15401 |
| 49 | PL-12 | Diarect | A15701 |
| 50 | PL-7 | Diarect | A15601 |
| 51 | PM/Scl-75 | Diarect | A17001 |
| 52 | Nucleolin | Diarect | A19701 |
| 53 | Ribo P0 | Diarect | A14101 |
| 54 | Ribo P1 | Diarect | A14201 |
| 55 | PDC-E2 | Diarect | A17901 |
| 56 | Ribo P2 | Diarect | A14301 |
| 57 | SRP54 | Diarect | A18401 |
| 58 | PM/Scl-100 | Diarect | A16001 |
| 59 | POLR3H | Origene | TP310633 |
| 60 | PDH | Sigma | P7032 |
| 62 | Ro60 | Diarect | A17401 |
| 65 | Scl-70 | Diarect | A12401 |
| 67 | Sm/RNP | Immunovision | SRC-3000 |
| 68 | Smith | Immunovision | SMA-3000 |
| 70 | Troponin I | Prospec | PRO-1269 |
| 72 | PDIA2 | MyBioSource | MBS1175372 |
| 73 | TG | Diarect | A12201 |
| 74 | GNAL | Abnova | H00002774-P01 |
| 75 | MYH6 | Origene | TP313673 |
| 76 | TPO | Diarect | A12101 |
| 77 | ZnT8 | UC Anschutz Medical Campus |  |
| 79 | U1-snRNP A | Diarect | A13101 |
| 80 | U1-snRNP C | Diarect | A13201 |
| 83 | RPP25 | Origene | TP303538 |

**Supplementary Table S2. Cytokine Array content.**

| **Bead ID** | **Antigen** | **Vendor** | **Catalog #** |
| --- | --- | --- | --- |
| 1 | Bare Bead |  |  |
| 2 | Human IgG from serum | Sigma | I4506 |
| 3 | Anti-Human IgG Fc fragment Specific | Jackson | 109-005-008 |
| 4 | Anti-Human IgG (H+L) | Jackson | 109-005-003 |
| 5 | Anti-Human IgG F(ab') fragment specific | Jackson | 109-005-006 |
| 6 | CD74 | Prospec | PRO-1467 |
| 7 | IFNλ2 | Peprotech | 300-02K |
| 8 | IL-1α | Prospec | CYT-253 |
| 10 | ITM2B | Elabscience | PKSH032599 |
| 26 | IL-31 | Prospec | CYT-625 |
| 27 | IL-6 | Prospec | CYT-098 |
| 29 | OSM | Peprotech | 300-10 |
| 30 | IL-11 | Prospec | CYT-214 |
| 32 | IL-27 | Prospec | CYT-048 |
| 33 | CNTF | Prospec | CYT-272 |
| 34 | CT-2 | Prospec | PRO-1578 |
| 35 | LIF | Peprotech | 300-05 |
| 36 | VEGFB | Peprotech | 100-20B |
| 37 | HTRA1 | R&D | 2916-SE-020 |
| 38 | GM-CSF | Peprotech | 300-03 |
| 39 | IFNα2 | R&D | 11101-2 |
| 40 | IFNβ | Peprotech | 300-02BC |
| 41 | IFNγ | Peprotech | 300-02 |
| 42 | IFNε | R&D | 9667-ME-025/CF |
| 43 | IFNλ1 | Peprotech | 300-02L |
| 44 | IFNλ3 | R&D | 5259-IL-025/CF |
| 45 | IFNω | R&D | 11395-1 |
| 46 | IL-10 | Peprotech | 200-10 |
| 47 | IL-12p40 | Peprotech | 200-12P40 |
| 48 | IL-12p70 | Peprotech | 200-12 |
| 49 | IL-15 | Peprotech | 200-15 |
| 50 | IL-17F | Peprotech | 200-25 |
| 51 | IL-1β | Peprotech | 200-01B |
| 52 | IL-22 | Peprotech | 200-22 |
| 55 | TNFα | Peprotech | 300-01A |
| 58 | ACE2 | Sino Biological | 10108-H05H |
| 59 | Eotaxin | Peprotech | 300-21 |
| 60 | Eotaxin 2 | Peprotech | 300-33 |
| 62 | IL-17A | Peprotech | 200-17 |
| 65 | MIP-1α | Peprotech | 300-08 |
| 67 | PDGFBB | Peprotech | 100-14B |
| 68 | sRANK-ligand | Peprotech | 310-01C |
| 69 | TIFIγ | Diarect | A11001 |

**Supplementary Table S3: Clinical Grade Autoantibody Assays**

| Clinical Assay | LabCorp Product # | Quest Product # |
| --- | --- | --- |
| Antinuclear Antibody (ANA) | 164855 | 249 |
| Anti-Ro | 520010 | 38568 |
| Anti-La | 012708 | 7832 |
| Anti-RNP | 006338 | 38567 |
| Anti-SM | 006338 | 37923 |
| Anti-dsDNA | 096339 | 255 |
| Anti-CCP | 164914 | 11173 |
| Direct Coomb’s Test | 006270 | 361 |

**Supplementary Table S4. Clinical-grade lab tests compared to the research-grade array assay.**

| **Sample** | **Pos AAb (array)** | **Anti-SSA (array)** | **Anti-SSA (clin)** | **Anti-SSB (array)** | **Anti-SSB (clin)** | **Anti-SM (array)** | **Anti-SM (clin)** | **Anti-RNP (array)** | **Anti-RNP (clin)** |
| --- | --- | --- | --- | --- | --- | --- | --- | --- | --- |
| UP01 R | * | NEG | NEG | NEG | NEG | NEG | * | NEG | * |
| UP01 F | * | NEG | NEG | NEG | NEG | NEG | * | NEG | * |
| UP02 R | Jo1 | NEG | NEG | NEG | NEG | NEG | * | NEG | * |
| UP02 F | Jo1 | NEG | NEG | NEG | NEG | NEG | * | NEG | * |
| UP03 R | * | NEG | NEG | NEG | NEG | NEG | NEG | NEG | NEG |
| UP03 F | * | NEG | NEG | NEG | NEG | NEG | NEG | NEG | NEG |
| UP05 R | * | NEG | NEG | NEG | * | NEG | * | NEG | * |
| UP05 F | * | NEG | NEG | NEG | * | NEG | * | NEG | * |
| UP06 R | * | NEG | * | NEG | * | NEG | * | NEG | * |
| UP06 F | * | NEG | * | NEG | * | NEG | * | NEG | * |
| UP07 F | * | NEG | * | NEG | * | NEG | * | NEG | * |
| UP08 R | Mi-2, Sm, Smith, PDC-E2 | NEG | * | NEG | * | POS | * | Undetermined | * |
| UP09 R | * | NEG | NEG | NEG | NEG | NEG | * | NEG | NEG |
| UP11 R | * | NEG | NEG | NEG | NEG | NEG | NEG | NEG | NEG |
| UP12 R | * | NEG | * | NEG | * | NEG | * | NEG | * |
| UP13 R | Ro60 | POS | POS | NEG | POS | NEG | NEG | NEG | NEG |
| UP14 R | SRP54 | NEG | * | NEG | * | NEG | * | NEG | * |
| UP15 R | * | NEG | NEG | NEG | * | NEG | NEG | NEG | * |
| UP17 R | CENP A, Smith, PCNA, GNAL | NEG | * | NEG | * | POS | * | NEG | * |
| UP18 R | * | NEG | * | NEG | * | NEG | * | NEG | * |
| UP19 R | * | NEG | * | NEG | * | NEG | * | NEG | * |
| UP21 R | CENP A | NEG | NEG | NEG | NEG | NEG | NEG | NEG | NEG |
| UP22 R | La | NEG | NEG | POS | NEG | NEG | NEG | NEG | NEG |
| UP24 F | Thyroglobulin | NEG | * | NEG | * | NEG | * | NEG | * |
| UP25 R | * | NEG | * | NEG | * | NEG | * | NEG | * |
| UP26 R | * | NEG | POS | NEG | NEG | NEG | NEG | NEG | NEG |
| UP27 F | * | NEG | * | NEG | * | NEG | * | NEG | * |
| UP28 R | * | NEG | * | NEG | * | NEG | * | NEG | * |
| UP29 R | La | NEG | * | POS | * | NEG | * | NEG | * |
| UP30 R | Ro60, La, SRP54 | POS | POS | POS | POS | NEG | NEG | NEG | NEG |
| UP31 R | * | NEG | * | NEG | * | NEG | * | NEG | * |
| UP33 F | * | NEG | * | NEG | * | NEG | * | NEG | * |
| UP34 F | * | NEG | * | NEG | NEG | NEG | * | NEG | * |
| UP35 F | * | NEG | * | NEG | * | NEG | * | NEG | * |
| **% Agree** |  | **94** | | **86** | | **100** | | **100** | |

**^** Thirty-four samples from 29 subjects in the UP cohort were screened by one or more clinical-grade lab tests. “POS” and “NEG” indicate positive and negative results, respectively, by clinical lab assay (clin) compared to the result on the custom bead-based array (array). Six patients had autoantibodies measured in both disease flare and remission, while a flare or remission sample, whichever available, was tested for the remaining 23 patients.

* indicates that a sample did not test positive for autoantibodies targeting any antigen on the CTD array or that the sample was not screened by a specific clinical lab test. The % agreement was calculated based on the amount of agreeing calls between the clinical and array based assays.

**Supplementary Table S5: Autoantibody Prevalence does not correlate with iMCD Clinical \Subtype in either the CTD (A) or ACA (B) arrays.** Autoantibody prevalence between iMCD clinical subtypes and other phenotypes. Number of positive and negative results in each array were quantified and a fisher’s exact test was used to determine significance between clinical subtypes. Significance was determined by a Bonferroni corrected *P*-value < 0.005.

1. **CTD Array**

| Group_1 | Group_2 | Pos_group1 | Neg_group1 | Pos_group2 | Neg_group2 | P-value |
| --- | --- | --- | --- | --- | --- | --- |
| TAFRO | NOS | 21 | 26 | 25 | 35 | 0.8 |
| TAFRO | IPL | 21 | 26 | 8 | 4 | 0.2 |
| TAFRO | DLBCL | 21 | 26 | 9 | 11 | 1 |
| TAFRO | HEALTHY | 21 | 26 | 5 | 25 | 0.01 |
| IPL | NOS | 8 | 4 | 25 | 35 | 0.13 |
| IPL | DLBCL | 8 | 4 | 10 | 10 | 0.29 |
| IPL | HEALTHY | 8 | 4 | 5 | 25 | **0.003*** |
| NOS | DLBCL | 25 | 35 | 10 | 10 | 0.8 |
| NOS | HEALTHY | 25 | 35 | 5 | 25 | 0.02 |
| DLBCL | HEALTHY | 9 | 11 | 5 | 25 | 0.051 |

1. **ACA Array**

| Group_1 | Group_2 | Pos_group1 | Neg_group1 | Pos_group2 | Neg_group2 | P-value |
| --- | --- | --- | --- | --- | --- | --- |
| TAFRO | NOS | 12 | 35 | 22 | 38 | 0.3 |
| TAFRO | IPL | 12 | 35 | 8 | 4 | 0.01 |
| TAFRO | DLBCL | 12 | 35 | 10 | 10 | 1 |
| TAFRO | HEALTHY | 12 | 35 | 3 | 27 | 0.14 |
| IPL | NOS | 8 | 4 | 22 | 38 | 0.11 |
| IPL | DLBCL | 8 | 4 | 10 | 10 | 0.47 |
| IPL | HEALTHY | 8 | 4 | 3 | 27 | **0.0005*** |
| NOS | DLBCL | 22 | 38 | 10 | 10 | 0.3 |
| NOS | HEALTHY | 22 | 38 | 3 | 27 | 0.01 |
| DLBCL | HEALTHY | 10 | 10 | 3 | 27 | **0.003*** |
